# A Novel Human TGF-β1 Fusion Protein in Combination with rhBMP-2 Increases Chondro-Osteogenic Differentiation of Bone Marrow Mesenchymal Stem Cells

**DOI:** 10.3390/ijms150711255

**Published:** 2014-06-25

**Authors:** Silvia Claros, Gustavo A. Rico-Llanos, José Becerra, José A. Andrades

**Affiliations:** 1 Laboratory of Bioengineering and Tissue Regeneration (LABRET), Department of Cell Biology, Genetics and Physiology, Faculty of Sciences, Universidad de Málaga, Campus de Teatinos, Málaga 29071, Spain; E-Mails: silviacg@uma.es (S.C.); gustavo.rico@gmail.com (G.A.R.-L.); becerra@uma.es (J.B.); 2 Networking Biomedical Research Center in Bioengineering, Biomaterials and Nanomedicine (CIBER-BBN), Madrid 28029, Spain; 3 BIONAND, Centro Andaluz de Nanomedicina y Biotecnología (Junta de Andalucía), Universidad de Málaga, Málaga 29590, Spain

**Keywords:** stem/progenitor cell, three-dimensional (3D) culture, transforming growth factor-beta1 (TGF-β1), bone morphogenetic protein-2 (BMP-2), osteogenesis, chondrogenesis

## Abstract

Transforming growth factor-beta (TGF-β) is involved in processes related to the differentiation and maturation of osteoprogenitor cells into osteoblasts. Rat bone marrow (BM) cells were cultured in a collagen-gel containing 0.5% fetal bovine serum (FBS) for 10 days in the presence of rhTGF (recombinant human TGF)-β1-F2, a fusion protein engineered to include a high-affinity collagen-binding decapeptide derived from von Willebrand factor. Subsequently, cells were moderately expanded in medium with 10% FBS for 4 days and treated with a short pulse of rhBMP (recombinant human bone morphogenetic protein)-2 for 4 h. During the last 2 days, dexamethasone and β-glycerophosphate were added to potentiate osteoinduction. Concomitant with an up-regulation of cell proliferation, DNA synthesis levels were determined. Polymerase chain reaction was performed to reveal the possible stemness of these cells. Osteogenic differentiation was evaluated in terms of alkaline phosphatase activity and mineralized matrix formation as well as by mRNA expression of osteogenic marker genes. Moreover*,* cells were placed inside diffusion chambers and implanted subcutaneously into the backs of adult rats for 4 weeks. Histological study provided evidence of cartilage and bone-like tissue formation. This experimental procedure is capable of selecting cell populations from BM that, in the presence of rhTGF-β1-F2 and rhBMP-2, achieve skeletogenic potential *in vitro* and *in vivo*.

## 1. Introduction

Adult stem cells from marrow stroma, operationally termed mesenchymal stem cells or marrow stromal cells (MSCs) [1,2], are now being considered for use in a wide range of tissue engineering technologies, and cell or gene therapy strategies, because of their high capacity for self-renewal [3,4], their multipotentiality for differentiation [5,6,7] and their demonstrable contributions to somatic tissue restoration [8,9,10]. With respect to the potential for clinical benefit in skeletal disorders, the possibility of using MSCs for bone tissue engineering has been suggested as an alternative strategy and a promising option, since the requirement for new bone in cases of bone loss caused by trauma, age and metabolic or genetic bone diseases is a major clinical and socioeconomic need [11,12].

To generate bone, MSCs need to undergo differentiation into the osteogenic lineage. Although the factors that regulate their *ex vivo* expansion and promote their osteogenic maturation remain poorly defined, it is now well established that members of the transforming growth factor-beta (TGF-β) family play a prominent role in the development, growth and maintenance of the vertebrate skeleton [13,14]. The effect of TGF-β1 on the proliferation and osteoblastic differentiation of MSCs *in vitro*—causing an increase in total cell number, alkaline phosphatase activity (ALP), and calcium content—is well documented [15,16,17]. Therefore, controlled administration of TGF-β1 could represent an emerging tissue engineering technology that may modulate cellular responses to encourage bone regeneration at a skeletal defect size [18,19].

In animal models of osteogenesis, TGF-β1 administration has been shown to stimulate osteoblast activity, causing the formation of new woven bone [14,20], and promoting the healing of fractures and skeletal defects [18,21]. It is important to note, however, that discrepant effects of TGF-β on proliferation and differentiation of MSC populations with osteogenic potential and bone formation have been reported [22]. Overall, the data emphasize that the physiological effects of TGF-β on cells of the osteoblast lineage appear to be highly complex and are influenced by the state of commitment and differentiation of the target cells, the cytokine milieu of the microenvironment, and the conditions of culture [22]. Thus, the effect of this growth factor is context-dependent and the apparent discrepant effects of TGF-β on proliferation and differentiation of cells with enhanced osteochondrogenic potential as well as on bone formation are probably owing to the manner in which it exerts its effects.

Bone morphogenetic proteins (BMPs) were originally identified by Urist as organic components found in bone matrix that could induce ectopic bone formation [23]. Of these, BMP-2 is one of the most potent osteoinductive cytokines which has been demonstrated to increase cartilage and bone formation in several animal models [24,25]. When cultures of MSCs are treated with BMP-2, these cells commit to the chondrogenic or osteogenic lineage and, depending on culture conditions, mature into either chondrocytes or osteoblasts. Furthermore, it has been reported that TGF-β1 in combination with BMP-2 strongly enhances ectopic bone formation, with the resulting bone volume being five-fold greater than that induced by BMP-2 alone [14,26].

Our laboratory has successfully developed a three-dimensional (3D) collagen gel culture system in the presence of a recombinant human TGF-β1 bearing a collagen-binding domain (rhTGF-β1-F2), in which adherent and non-adherent bone marrow (BM) stem cells are selected and expanded in order to obtain cells capable to differentiate into bone and cartilage [27,28,29,30]. Differences between rhTGF-β1-F2 and the commercial rhTGF-β1 may be due to the slow release of the collagen-binding factor from the collagen fibrillar network to which it is bound, resulting in a longer half-life [27]. Therefore, our previous results suggested that rhTGF-β1-F2, when applied to a collagen matrix as a vehicle and delivery system, could be of advantage in promoting survival, proliferation, and differentiation and colony mineralization of the osteogenic precursor cell population.

The goal of the present study was to complete the characterization of rat BM-derived cells in the 3D culture system and analyze the effects of a pulse of rhBMP-2 for only 4 h in the chondro-osteogenic differentiation of these cells. We evaluated the expression of mesenchymal, hematopoietic, endothelial and osteogenic-specific markers, ALP activity and calcium content *in vitro*, and their capacity to differentiate into cartilage and bone tissue when they are ectopically implanted *in vivo*. Improvement of our 3D collagen culture may lead to isolation, expansion and differentiation of a heterogeneous population of BM-derived cells for use in tissue engineering and regenerative medicine applications.

## 2. Results and Discussion

### 2.1. Morphology and Cell Number in 3D (Three-Dimensional) Collagen Culture

**Figure 1:**
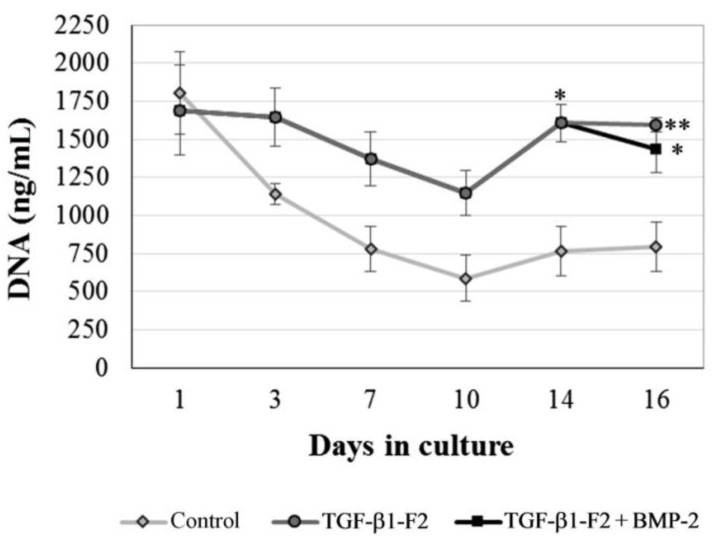
Quantification of DNA content as indication of cell replication. Values represent the means ± SD, *n* = 3; * *p* < 0.05, ** *p* < 0.01.

3D culture was formed by a population of smaller round cells evenly distributed and embedded in collagen matrix; the number of cells sharply decreasedc as a consequence of the starvation period for the initial 10 days (Figure 1). Once normal serum conditions were reestablished, the selected cells began to proliferate either in the presence or absence of rhTGF-β1-F2. Nevertheless, the number of cells harvested from rhTGF-β1-F2-treated cultures at day 14 and 16 was significantly higher (*p* < 0.05 and *p* < 0.01, respectively) when compared with controls. In rhTGF-β1-F2-treated cultures induced also with a pulse of rhBMP-2 on day 14 slightly lower cell numbers were obtained, only significantly different compared to control group (*p* < 0.05 at day 16).

**Figure 2:**
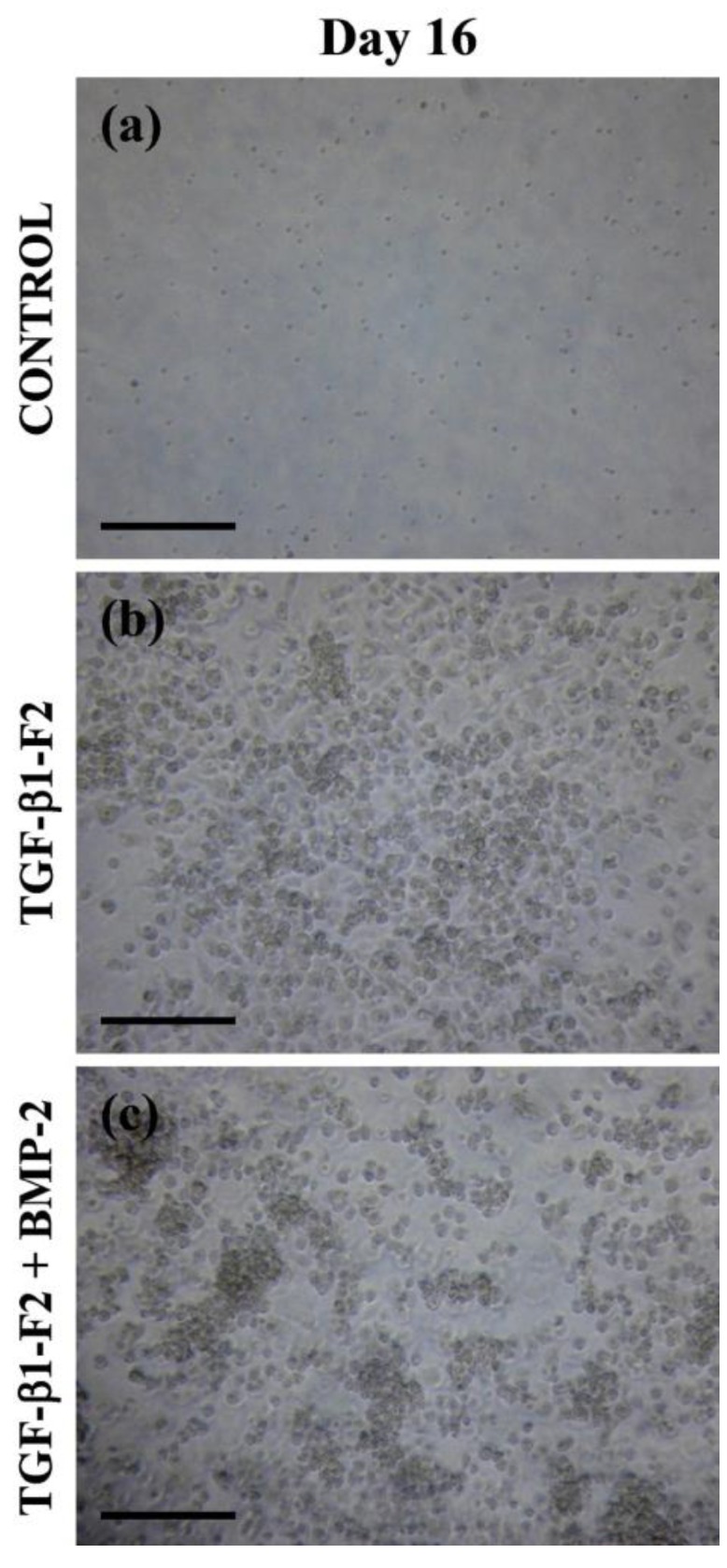
Cell morphology in 3D (three-dimensional) collagen matrix at the end of differentiation period (day 16). (**a**) Control cultures in the absence of growth factor; (**b**) Cell cultures in presence of rhTGF-β1-F2; and (**c**) Cell cultures in presence of rhTGF (recombinant human transforming growth factor)-β1-F2 and the pulse of rhBMP (recombinant human bone morphogenetic protein)-2. Bars, 200 μm.

Under a phase contrast microscope, these cells cultured with rhTGF-β1-F2 (in absence or presence of rhBMP-2) increased in size and formed well-defined colonies at the end of differentiation period (Figure 2). BM-derived cells cultured under control conditions never formed colonies and presented a different morphology.

### 2.2. Flow Cytometry Analysis of Cells

**Figure 3:**
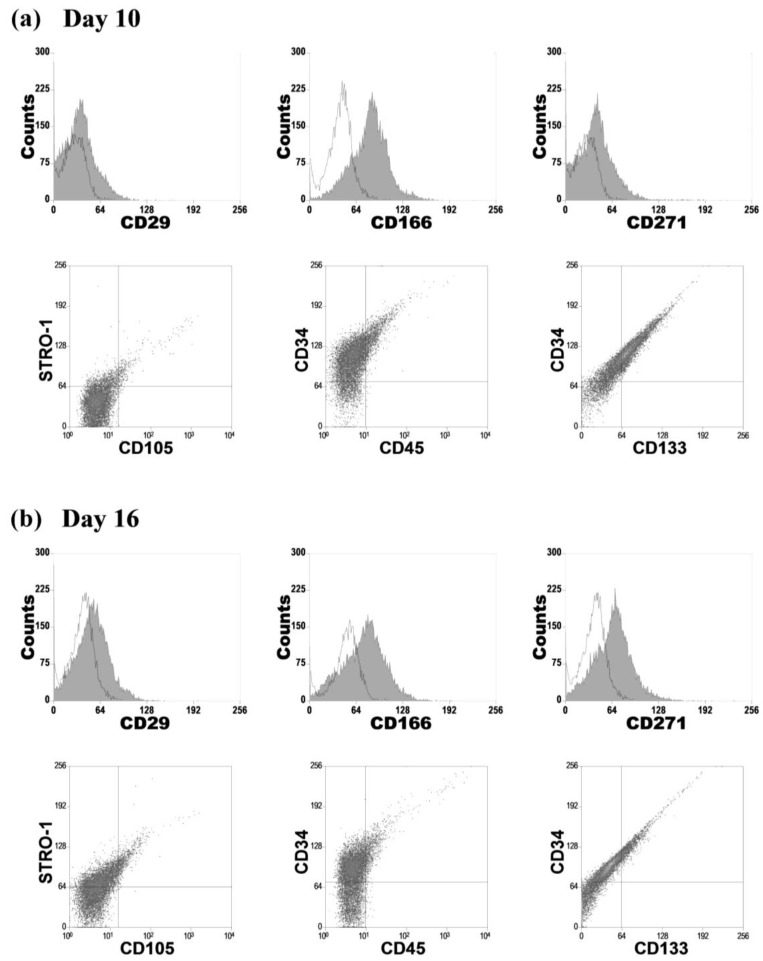
Immunophenotype profiles of 3D culture cells for hematopoietic, mesenchymal and endothelial markers. Representative FACS (fluorescence-activated cell sorting) analysis at days 10 (**a**) and 16 (**b**).

ADH (Adherent culture) culture showed the immunophenotype described for MSCs, where most of the cells expressed CD29, CD105, CD166, CD271 and STRO-1, and were negative for CD34, CD45 (data not shown). However, the profiles of 3D culture revealed a heterogeneous cell population, where positive cells for hematopoietic (CD34, CD45), mesenchymal (CD29, CD105, CD166, CD271, STRO-1), and endothelial markers (CD34, CD133) were found (Figure 3). Comparing 3D cultures at days 10 and 16, CD166+ population decreased over time (17.5%, *p* < 0.05), while there was an increase in the expression of some mesenchymal cell markers such as CD29, CD271 and STRO-1 (+29.2%, +25.1%, +39.3%, *p* < 0.001, respectively). In addition, the endothelial progenitor cells (EPCs)-like population (CD34+/CD133+/CD45−) decreased significantly (−14.8%, *p* < 0.001).

### 2.3. Quantitative Real Time RT-PCR (RT-qPCR) Analysis

**Figure 4:**
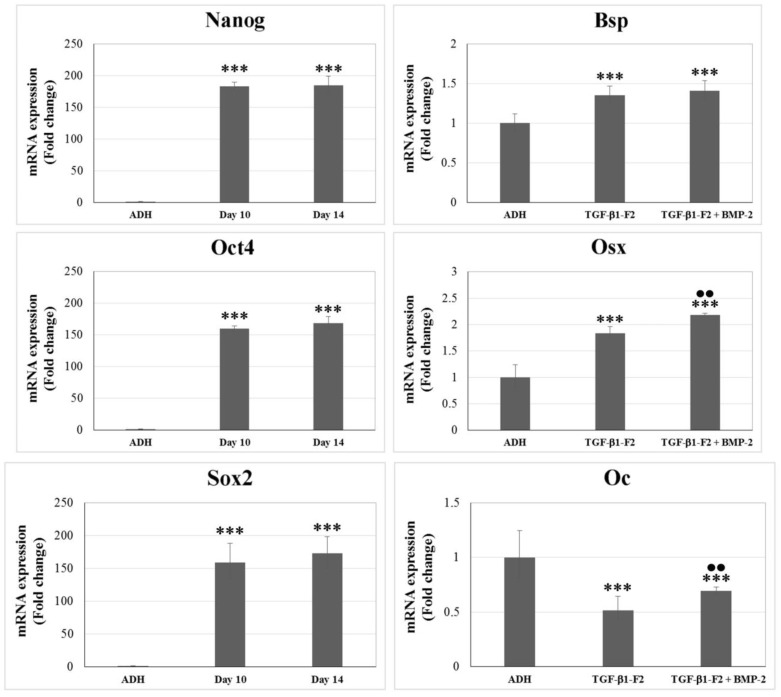
mRNA expression in 3D and ADH (adherent culture) cultures by quantitative real time RT-PCR (RT-qPCR). rhTGF-β1-F2-treated cells expressed Nanog, Oct4 and Sox2 at the end of selection and amplification period (days 10 and 14, respectively), while ADH cells did not. Moreover, the osteogenic commitment of 3D culture cells at the end of the differentiation period (day 16) was confirmed by the mRNA expression of Bsp (Bone sialoprotein), Osx (Osterix) and Oc (Osteocalcin). The pulse of rhBMP-2 significantly induced further differentiation towards the osteogenic lineage. Values are the means ± SD, *n* = 3; *** *p* < 0.001 *vs.* ADH culture, •• *p* < 0.001 *vs.* rhTGF-β1-F2.

Pluripotency of embryonic stem cells is controlled by defined transcription factors, such as Nanog, Oct4 and Sox2. We analyzed the expression of mRNA for these pluripotent specific markers by RT-qPCR at days 10 and 14, and the signal was compared to ADH culture (Figure 4). Nanog, Oct4 and Sox2 were highly expressed in both 3D cultures at days 10 and 14 but was absent in ADH cells (*p* < 0.001). In addition, we evaluated the osteoblastic potential of 3D culture cells at the end of the differentiation period (day 16), analyzing the expression of mRNA Bsp (Bone sialoprotein), Osx (Osterix) and Oc (Osteocalcin) (Figure 4). Bsp and Osx mRNA displayed the highest expression levels in 3D cultures (*p* < 0.001 *vs.* ADH). Oc mRNA was also detected in 3D culture cells, but its expression level was significantly lower than that found in ADH cells (*p* < 0.001). Comparison within the 3D culture showed that Osx and Oc mRNA levels were significantly different with or without the pulse of rhBMP-2 (*p* < 0.01). Thus, the osteoblast phenotype was revealed by demonstrating the expression of Bsp, Osx and Oc genes involved in the osteogenic lineage.

### 2.4. Biochemical Assays

**Figure 5:**
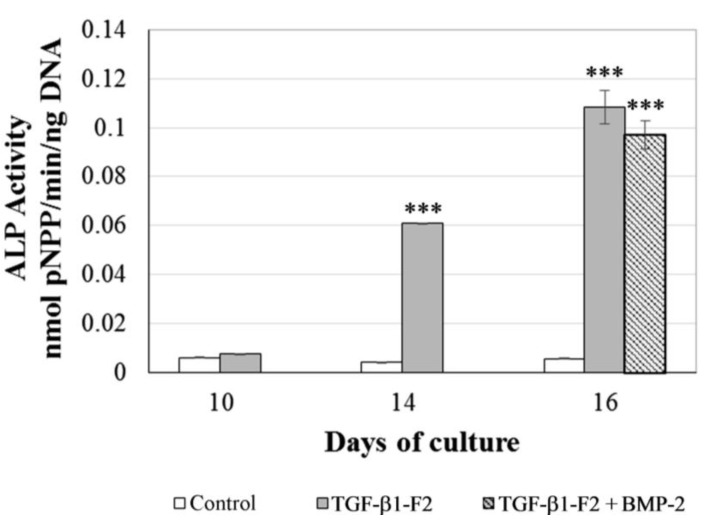
Effects of culture conditions on ALP (alkaline phosphatase activity) activity. Values represent the means ± SD, *n* = 3; *** *p* < 0.001. pNPP: *p*-nitrophenolphosphate.

Bone cell differentiation was further confirmed by measuring the ALP specific activity and the amount of calcium deposited. Positive ALP expression was detected only in induced cells, which exhibited significantly higher levels (*p* < 0.001) compared with the controls (Figure 5). At day 14, there were a 14.5-fold difference between control and rhTGF-β1-F2-treated cells which rises to 19.3-fold at day 16. Similar levels of ALP activity were observed in cells incubated with rhTGF-β1-F2 and rhBMP-2 (17.4-fold *vs.* control group at day 16).

Likewise, an important increase in calcium deposition was detected in rhTGF-β1-F2-treated cells at day 16 (1.74 ± 0.25 mg/dL, *p* < 0.01) (Table 1), which was significantly higher in cultures induced also with a pulse of rhBMP-2 (2.47 ± 0.21 mg/dL, *p* < 0.001 *vs.* control and *p* < 0.05 *vs.* rhTGF-β1-F2-treated cells).

**Table 1:** Quantification of calcium content as indication of mineralization.

Time (Days)	Treatment	Calcium (mg/dL)
10	Control	0.41 ± 0.26
	TGF-β1-F2	0.62 ± 0.37
14	Control	0.47 ± 0.16
	TGF-β1-F2	0.71 ± 0.23
16	Control	0.50 ± 0.16
	TGF-β1-F2	1.74 ± 0.25 **
	TGF-β1-F2 + BMP-2	2.47 ± 0.21 ***

Values are expressed as the mean ± SD (*n* = 3). ** *p* < 0.01, *** *p* < 0.001.

### 2.5. In Vivo Implantation and Histological Study

**Figure 6:**
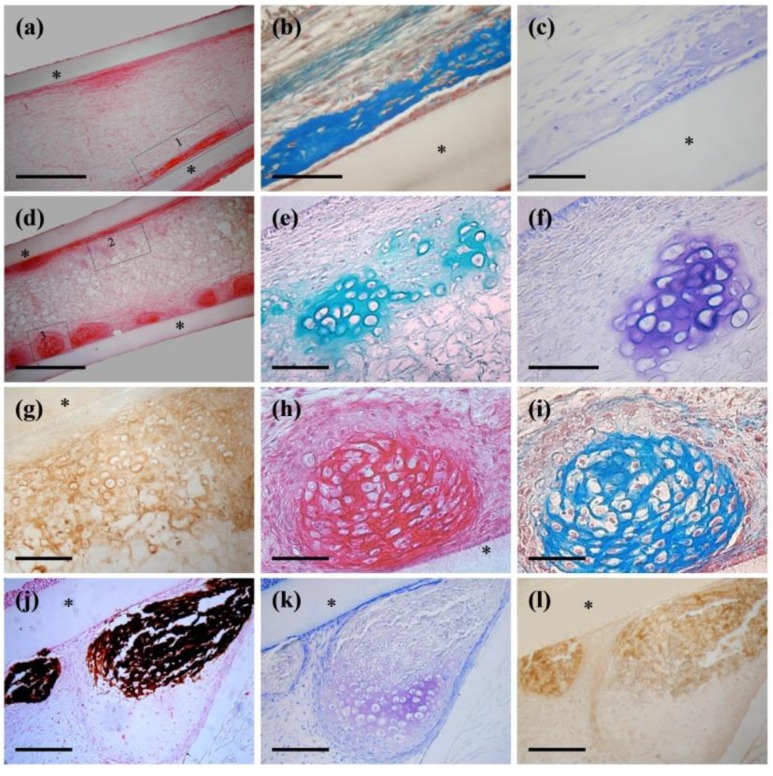
Histological sections of diffusion chambers with 3D culture cells after 4 weeks of *in vivo* implantation. Different types of tissue can be observed, according to cell type implanted. Asterisks (*) represent filters of the chambers. (**a**–**c**) rhTGF-β1-F2-treated cultures induced with a pulse of rhBMP-2. *In vivo* bone-like tissue formation was demonstrated. (**a**) PSH (picrosirius-hematoxyline); (**b**–**c**, details from zone 1) Goldner’s trichrome and TB (toluidine blue) stain; (**d**–**l**) rhTGF-β1-F2 and rhBMP-2-treated culture cells. Several nodules of cartilage and bone-like tissue located near the filters were observed. (**d**) PSH; (**e**–**g**, details from zone 2) AB (alcian blue), TB and type II collagen immunohistochemistry; (**h**–**i**, details from zone 3) PSH and Goldner’s trichrome stain; (**j**) von Kossa stain; (**k**) TB; (**l**) type I collagen immunohistochemistry. Bars, 500 μm in (**a**,**d**), 200 μm in (**b**,**c**,**e**–**i**) and 100 μm in (**j**–**l**).

One million of 3D cells were implanted into diffusion chambers and harvested after 4 weeks. Histological analysis revealed that control implants were principally filled with loose fibrous connective tissue. Importantly, neither cartilage nor bone is formed in these chambers (data not shown). In contrast, *in vivo* bone-like tissue formation was demonstrated in rhTGF-β1-F2 treated cultures (Figure 6a–c). Sections stained with PSH (picrosirius-hematoxyline) (Figure 6a) and Goldner’s trichrome (Figure 6b, detail from zone 1) showed dense tissue condensations with osseous matrices localized adjacent to the Millipore filter. Figure 6c show parallel section with negative area for TB (toluidine blue). However, the best results were obtained in chambers with cells induced with rhTGF-β1-F2 and rhBMP-2 (Figure 6d–l). We found several nodules of cartilage and bone-like tissue located near the filters. Cartilage nodules (zone 2) displayed metachromasia when stained with TB (Figure 6e), showed high affinity for AB (alcian blue) (demonstrating the presence of sulfated glycosaminoglycans) (Figure 6f) and were collagen II immunoreactive (Figure 6g), indicating a cartilaginous matrix. In contrast, bone-like condensations near the wall of the chamber (Figure 6d, zone 3), stained positively with PSH (Figure 6h) and Goldner’s trichrome (Figure 6i). We normally distinguished the bone condensation close to the filter, as demonstrated by von Kossa staining (Figure 6j) and type I collagen immunohistochemistry (Figure 6l), while cartilage is formed towards the centre of the chamber (metachromasia with TB in Figure 6k).

### 2.6. Discussion

The vast majority of *in vitro* studies in the regenerative medicine and tissue engineering field are performed in 2-dimensional structures. Researchers have expressed the necessity to develop culture systems that better represent the natural environment of cells in tissues and organs. Therefore, our group has successfully developed a 3D collagen gel culture system [27,28,29,30] that allows one to study the isolation, expansion and growth factor responsiveness of BM-derived cells and evaluate the capacity of these cells to undergo differentiation into bone and/or cartilage tissue when implanted *in vivo*.

In the present study we sought to characterize further BM-derived cells, isolated in the described 3D collagen gel culture system and exposed to a novel recombinant human TGF-β1 fusion protein (rhTGF-β1-F2), engineered to contain an auxiliary collagen-binding domain. Besides, we investigated whether the combination of rhTGF-β1-F2 and rhBMP-2 enhances the differentiation of MSCs towards the chondro-osteogenic lineage.

After the 10-day selection period, the surviving cells in the rhTGF-β1-F2 test groups proliferated rapidly in response to serum factors, and maximal DNA synthesis levels were observed. Upon the addition of rhBMP-2 and osteoinductive factors, proliferation was evidently downregulated, which is associated with expression of osteoblast functions, including production of ALP, processing of procollagens to collagens, and incremental deposition of a collagenous extracellular matrix [31,32]. As a result, it is widely accepted that high expression of ALP and production of a mineralized matrix serve as useful markers of osteogenesis, where the mineralization marks the final phase of osteoblast phenotypic development [33,34,35]. Previous reports by our group demonstrated that there was an interesting difference in results between the commercial and modified rhTGF-β1 [27]. Only rhTGF-β1-F2-treated cells were able to express higher levels of ALP activity and to induce calcium precipitation. Here, the pulse of rhBMP-2 at day 14 significantly increased calcium deposition compared to other conditions. Moreover, this result was consistent with the mineralization of colonies observed in these cultures. All this suggests that the pulse of rhBMP-2 of 4 h at the end of the proliferation period incorporates an obvious advantage in differentiation and colony mineralization of the osteogenic precursor cell population.

We also analyzed the expression of surface molecule characteristic for mesenchymal, endothelial and hematopoietic lineages using FACS. 3D culture revealed a heterogeneous population of small round cells [36,37]. These results were consistent with our previous studies with human BM-derived cells and commercial rhTGF-β1 in the 3D culture [36], although the immunophenotyping was difficult because of the limited availability of antibodies that show cross-reactivity with the rat. The study of the surface marker profile showed positive cells for hematopoietic (CD34, CD45), mesenchymal (CD29, CD105, CD166, CD271, STRO-1) and endothelial markers (CD34, CD133) [38,39,40,41,42]. Comparing 3D cultures at days 10 and 16, we observed an increase in the expression of mesenchymal cell markers, while EPCs-like populations (CD34+/CD133+/CD45−) significantly decreased over time. Furthermore, our previous results demonstrated that EPCs-like populations decreased in favor of mature endothelial phenotypes (CD146+/KDR+) [43,44], demonstrating that our 3D culture system could be used as a strategy to promote angiogenesis and to improve bone regeneration [36,45,46].

Additionally, we evaluated the expression of pluripotent specific markers by RT-qPCR. Results showed that Nanog, Oct4 and Sox2 were significantly highly expressed in 3D culture at days 10 and 14 but was absent in ADH cells. These findings are also in agreement with those previously reported by our group [36]. Nanog, Oct4 and Sox2 target genes, identified in embryonic stem cells (ESCs), are known to overlap substantially, suggesting they collaborate to regulate a common set of genes governing pluripotency, self-renewal, and cell fate determination [47,48,49,50,51]. Thus, it demonstrated that induced expression of Nanog, Oct4 and Sox2 along or not with other regulatory proteins is enough to reprogram lineage-restricted somatic cell populations [52,53]. These cells, which were called iPS (induced pluripotent stem) cells, exhibit the morphology and growth properties of ESCs and express ESC marker genes [54,55,56].

For the first time, this study also provided mRNA expression data of genes involved in the osteogenic lineage, as were Bsp, Osx and Oc. Our results confirmed the osteogenic commitment of rhTGF-β1-F2-treated cells, and that the pulse of rhBMP-2 significantly increased mRNA levels of Osx and Oc. Osx is an osteoblast-specific transcription factor required for bone formation and mineralization *in vivo*. In Osx knock-out mice, there is no endochondral or intramembranous bone formation. Moreover, it is known that Osx is essential for the expression of others osteoblast-specific markers such as Bsp, type I collagen, Oc, osteonectin, and osteopontin [56,57,58].

Taken together, these facts suggest that rhTGF-β1-F2 applied to a collagen matrix as vehicle and delivery system could be of advantage in promoting the survival, proliferation, differentiation, and colony mineralization of the osteogenic precursor cell population. The pulse of rhBMP-2 seemed to be beneficial for inducing further differentiation of BM-MSCs towards the osteogenic lineage.

Histological study of 3D culture cells showed significant differences regarding the histogenesis that occurs inside the chambers, depending on the treatment. On the one hand, rhTGF-β1-F2-treated cells were only able to generate new bone, which seems to have intramembranous origin because no trace of cartilage can be found, such as hypertrophic chondrocytes, cartilage in the process of calcification, or remnants of proteoglycans [1,59]. Nevertheless, rhTGF-β1-F2 and rhBMP-2-treated cells formed abundant cartilage and bone-like nodules, some of them developed via endochondral ossification [59,60]. On the other hand, we normally observed cartilage nodules towards the center of the chambers and bone accumulation close to the filter, in agreement with other studies [61,62,63,64]. This could be related to the nutritional situation of the different regions of the chambers.

The *in vivo* analysis proves the efficiency of rhTGF-β1-F2 in selecting a population of precursor cells with chondro-osteogenic potential, and the important role of BMP-2 in the differentiation period in inducing cartilage and bone formation.

## 3. Experimental Section

### 3.1. Recombinant Human TGF (Transforming Growth Factor)-β1 Fusion Protein

The full coding region of the rhTGF-β1 fusion protein, rhTGF-β1-F2, was generously provided by ME Nimni (University of Southern California, Los Angeles, CA, USA), and details of the technique have been described elsewhere [27,28,29,65]. Briefly, the cDNA sequence encoding the conserved carboxy-terminal region of rhTGF-β1 was engineered to include a high-affinity collagen-binding decapeptide derived from von Willebrand factor bracketed by strategic linkers in frame with an *N*-terminal 6× His purification tag provided by an expression vector.

### 3.2. Isolation of Primary MSCs (Marrow Stromal Cells) and 3D Culture

Primary cultures of MSCs were established using BM suspensions from rat origin. Rat BM-derived cells were extracted from femurs and tibiae of syngeneic 8-week-old male Fisher 344 rats (Charles River Laboratories, L’Abresles, France) under animal care procedures, conducted in accordance with the guidelines set by the European Community Council Directives (86/609/EEC) and approved by the local ethical committee. The epiphysis was removed and the medullar canal was washed with Dulbecco’s modified Eagle’s medium (DMEM, Sigma-Aldrich, Madrid, Spain) using a syringe. The medium containing the extracted cells was directed into a Falcon tube (Becton Dickinson, Barcelona, Spain) and the cells were suspended, homogenized, and centrifuged at 400 g for 10 min.

BM-derived cells were cultured in a 3D environment using a modification of a previously described method [27]. Briefly, a collagen substrate for cell culture was prepared using a solution containing 0.85 mg/mL rat tail tendon type I collagen (Becton Dickinson), 1 M NaOH, 10× DMEM-F12, 100 U/mL penicillin, 100 μg/mL streptomycin and 1.25 μg/mL fungizone (all from Sigma-Aldrich) in MilliQ water at pH 7.4. 48-well plates (Nunc, Thermo Fischer Scientific, Hennigsdorf, Germany) were coated with 150 μL of this solution and placed at 37 °C for 30 min in order for it to solidify into a thin collagen gel matrix. After that, all of the cells were re-suspended in DMEM-F12 and mixed with the collagen solution plus 0.5% FBS, in the presence of 1 ng/mL rhTGF-β1-F2 at a density of 2.5 × 10^5^ cells/250 μL collagen/well in 48-well plates. The culture plates were left 30 min at 37 °C to allow the collagen to gel. Then, 250 μL/ well of culture medium that consisted of DMEM supplemented with 1 ng/mL rhTGF-β1-F2, 0.5% FBS, 2.5 mM l-Glutamine (both from Sigma-Aldrich) and the same amount of penicillin-streptomycin and fungizone as described above were added on top of the gel. Cells were incubated in this culture medium containing 0.5% FBS for 10 days in order to eliminate hematopoietic cells. After the 10-day selection period, the medium was then changed to 10% FBS and cells were cultured for an additional 4 days (amplification period). Afterwards, cells were treated with a short pulse of recombinant human BMP-2 (rhBMP-2, R&D Systems Europe, Abingdon, UK) for 4 h. At the end of the culture, cells were incubated for 2 days in medium supplemented with 10^−^^8^ M Dexametasone (Dex) and 2 mM β-glycerophosphate (β-GP) (both from Sigma-Aldrich) to help with the osteogenic differentiation. Control cultures were maintained without adding growth factor. Cultures were fed every third day with appropriate fresh medium and maintained at 37 °C in a humidified atmosphere containing 95% air and 5% CO_2_.

Adherent culture (ADH) was used like a control in RT-qPCR and flow cytometry analysis. BM-derived cells were plated at a concentration of 10^7^ cells/75-cm^2^ tissue culture flask and maintained in DMEM containing 10% FBS, 2.5 mM l-glutamine, 100 U/mL penicillin, 100 μg/mL streptomycin, and 1.25 μg/mL fungizone. The culture medium was changed two times per week, and the cells were selected by their capacity to attach to the dish surface, discarding the floating cells in the first medium change at 72 h. When culture flasks became near-confluent, cells were detached with 0.25% trypsin containing 1 mmol/L EDTA and subsequently replated at 5 × 10^3^ cells/cm^2^ for continued passaging.

### 3.3. Flow Cytometry Analysis of Cells

In order to analyze the expression of surface markers characteristic for mesenchymal, endothelial and hematopoietic cells, fluorescence-activated cell sorting (FACS) was performed at days 10 and 16, using specific fluorochrome-conjugated monoclonal antibodies. Rat BM-derived cells were washed twice in FACS buffer consisting of 10 mM hepes (Gibco^®^/Invitrogen, Barcelona, Spain), 100 U/mL penicillin, 100 μg/mL streptomycin and 2 mg/mL bovine serum albumin (Sigma, Madrid, Spain) in Leibovitz’s L-15 medium (Gibco). After the washing step, cells aliquots (1 × 10^6^ cells) were incubated in FACS buffer containing monoclonal antibodies against CD29 (BD Pharmigen, Becton Dickinson, Barcelona, Spain), CD34, CD45, CD133 and CD271 (Miltenyi Biotech, Madrid, Spain), CD105 and STRO-1 (R&D Systems) and CD166 (AbD Serotec, Oxford, UK), or an appropriate isotype control antibody (Sigma-Aldrich). After 30 min in the dark on ice, cells were washed again in FACS buffer before flow cytometry analysis. One hundred thousand events per sample were analyzed on a MoFlo^®^ SP1338 (DakoCytomation, Glostrup, Denmark) using Summit software. Cells were gated on forward and side scatter to exclude debris and cell aggregates, and dead cells were excluded by 7-Amino-Actinomycin D (7-AAD, BD Pharmigen) staining.

### 3.4. RT-qPCR Analysis

The expression levels of rat pluripotency genes Nanog, Oct4 and Sox2 were analyzed for 3D cultures after 10 and 14 days by RT-qPCR. Moreover, the osteogenesis markers Bsp, Osx and Oc were evaluated at the end of differentiation period (day 16). Reference gene *Gapdh* was used as positive control. Total RNA was isolated using RNeasy Kit (Qiagen, Valencia, CA, USA) and its quantity and purity were estimated by Nanodrop (Thermo Fischer Scientific). Only samples with an A260/A280 nm ratio between 1.8 and 2.0 were accepted. 1 μg of the total RNA sample was used as a template for cDNA synthesis by SuperScript^®^ First-Strand Synthesis System Kit and controls “minus RT” were performed to test possible genomic DNA amplification (Invitrogen, Carlsbad, CA, USA). Both protocols were performed following the manufacturer’s directions. Primer sequences, accession numbers for rat mRNA and references papers are in Table 2. All primer sequences were designed using Oligo Explorer 1.5 software (GeneLink™, Hawthorne, NY, USA) and determined through established GenBank sequences.

**Table 2:** Primer sequences for RT-qPCR with the expected product size.

Gene Name	Accession Number	Primer Sequence 5'-3' (Forward, Reverse) Reference	Theoric/Used Annealing Temperature (°C)	Product Size (bp)
Nanog	NM_001100781	GCCCTGAGAAGAAAGAAGAGAA TACCTTTGCCTCTGAAACCT	59.3/60.0	112
Oct4 (Pou5f1)	NM_001009178	CCCATTTCACCACACTCTACTC GACAGGAACAGAGGGAAAGG Han *et al*. 2012 [66]	60.9/60.0	68
Sox2	NM_001109181	ATTACCCGCAGCAAAATGAC GCGTTAATTTGGATGGGATTGG Han *et al*. 2012 [66]	59.0/60.0	60
Bsp (Ibsp)	NM_012587	ATGAAGGAAAGCGACGAGGA GTGGAGTTGGTGCTGGTG	60.9/60.0	113
Osx (Sp7)	NM_001037632	CTTTCCCCACTCATTTCCTG CTAGGCAGGCAGTCAGAAG Takahashi *et al*. 2008 [67]	61.1/60.0	90
Oc (Bglap)	NM_013414	ACCCTCTCTCTGCTCACTC CTTACTGCCCTCCTGCTT Zhou *et al*. 2010 [68]	61.8/60.0	124
Gapdh	NM_017008	CATGCCGCCTGGAGAAAC CCCAGGATGCCCTTTAGT Han *et al*. 2012 [66]	60.9/60.0	88

RT-qPCR was done with SYBR^®^ Premix Ex Taq™ following the manufacturer guidelines (Takara Bio, Inc., Otsu, Japan). Amplifications were performed and monitored in a 7500 Real-Time PCR System (Applied Biosystems, Madrid, Spain). Optimal quantities of each primer and cDNA dilution were tested for every gene and sample. The PCR amplification conditions were: 10 s at 95 °C of an initial denaturation, 40 cycles consisting of 3 s at 95 °C and 25 s at 60 °C. Each cDNA sequence was tested in triplicate and a dissociation melt curve protocol was run after every PCR reaction to determine the specificity of products. The levels of gene expression were determined by the comparative *C*_t_ method normalized with the endogenous control (Gapdh, glyceraldehyde 3-phosphate dehydrogenase), and compared to those in ADH cells.

### 3.5. Biochemical Assays

After 10, 14 and 16 days in culture, quantitative measurements of cell number (expressed as DNA amount), ALP activity and calcium content were determined. For determination of DNA amount and ALP activity, cells were lysed with 0.15 M NaCl, 3 mM NaHCO_3_ and 0.1% Triton X-100 (all from Sigma-Aldrich, St. Louis, MO, USA) in distilled water, pH 7.4, and repeatedly frozen-thawed for three times to disrupt the cell membranes. After that, the cells were sonicated for 30 s using an ultrasonic homogenizer (Vibra-Cell sonicator, Sonics and Materials, Inc., Newtown, CT, USA). DNA amount was determined by incubating 100 μL of cell lysates with 100 μL of Hoechst 33285 solution (1 μg/mL) (Sigma-Aldrich). Hoechst 33285 intercalates with DNA and emits fluorescence (maximum at 460 nm) when excited with ultraviolet light. Fluorochrome emission was measured at 450 nm by a fluorescence microplate reader (FL600, Bio-Tek Instruments, Winooski, VT, USA). DNA was determined in reference to a standard curve generated from serial-diluted DNA activated from calf thymus (Sigma-Aldrich). For analysis of ALP activity, aliquots of cell lysates were incubated with p-nitrophenolphosphate (pNPP, Sigma-Aldrich) for 30 min at 37 °C, and the results were quantified at 405 nm using a microplate reader (EL × 800, Bio-Tek Instruments). For determination of calcium levels, the cells were washed with Ca^2+^ and Mg^2+^ free PBS and then solubilized with 0.6 N HCl (both from Sigma-Aldrich). Measurements were done by colorimetry, using the 587-A calcium assay kit (Sigma-Aldrich), and the colorimetric reaction was read at 570 nm. The absolute calcium concentration was determined according to a standard curve according to the manufacturer’s recommendations. All assays were determined in triplicate for each condition and the data are expressed as mean ± SD.

### 3.6. In Vivo Implantation and Histological Study

One million cells were inoculated into diffusion chambers (150 μL volume. Commercial discs were made of a plastic ring and two Millipore filters of 0.45 μm pore size, all from Millipore, Madrid, Spain) and then implanted subcutaneously into syngeneic 10-week-old Fisher 344 rats under anesthesia. Chambers were harvested 4 weeks after implantation and processed for histology. Briefly, they were fixed in Bouin’s solution (Panreac, Barcelona, Spain) or 4% paraformaldehyde (Sigma-Aldrich) and dehydrated in alcohol, embedded in paraffin (Panreac, Barcelona, Spain) and sectioned at 8 μm thick. Afterwards, sections were stained with picrosirius-hematoxyline (PSH, Panreac), a technique that shows specificity for type I collagen, alcian blue (AB, Sigma-Aldrich), that reveals glycosaminaglycans from the cartilage matrix, toluidine blue (TB, Panreac) that exhibits metachromatic reaction with cartilage matrix, Goldner’s trichromic (Sigma-Aldrich) that stains mineralized type I collagen fibers and von Kossa (Sigma-Aldrich) that reveals calcium phosphate deposits in black. Additionally, consecutive sections were analysed immunohistochemically for type I and II collagen.

For immunohistochemical analyses, deparaffinised sections were rehydrated and then endogenous peroxidase was blocked with 3% hydrogen peroxide (Panreac). For type II collagen detection, sections were digested with papain at 0.5 mg/mL in phosphate buffer (pH 4.7) for 15 min at 37 °C (both from Panreac). Afterwards, they were incubated with rabbit polyclonal anti-collagen type I and II antibody (Calbiochem-Novabiochem Co., San Diego, CA, USA) at a 1:60 and 1:200 dilution, respectively, in buffer consisting of Tris-PBS pH 7.8, 0.5% Triton X-100, 1% BSA and 5% sheep serum (all from Sigma-Aldrich) overnight at 4 °C, followed by incubation with goat anti-rabbit IgG (Sigma-Aldrich) diluted 1:50 for 1 h, and PAP-rabbit complex (Dako) diluted 1:200 for 1 h. All incubations were carried out in a humid chamber and sections were washed three times for 5 min in Tris-PBS between each stage. The PAP complex was visualized by 3,3'-diaminobenzidine tetrahydrochloride (DAB, Sigma-Aldrich). After rinsing in distilled water, sections were dehydrated in ascending ethanol solutions, cleared in xylene and mounted. Control sections were incubated without the primary antibody.

### 3.7. Statistics

Means and standard deviations were performed using Sigma Stat software (SPSS Inc., Chicago, IL, USA) with analysis of variance (ANOVA), followed of Tukey post hoc test, after the data passed normality and equal variance tests. Results were considered significantly different at *p* > 0.05.

## 4. Conclusions

In conclusion, our results support that rhTGF-β1-F2 is a highly promising factor for expanding BM-derived cells maintaining their properties of stemness during the 10-day selection period and improving their chondrogenic and osteogenic differentiation. The pulse of rhBMP-2 for only 4 h incorporated a significant advantage in increasing the amount of cartilaginous and bone tissue *in vivo*. Therefore, BM-derived cells cultured in the 3D collagen matrix in the presence of rhTGF-β1-F2 and rhBMP-2 could be an important therapeutic tool for tissue engineering and regenerative medicine.
